# Fibrosis regression is induced by AdhMMP8 in a murine model of chronic kidney injury

**DOI:** 10.1371/journal.pone.0243307

**Published:** 2020-12-04

**Authors:** Homero Contreras-Salinas, Alejandra Meza-Rios, Jesús García-Bañuelos, Ana Sandoval-Rodriguez, Laura Sanchez-Orozco, Leonel García-Benavides, Ricardo De la Rosa-Bibiano, Hugo Christian Monroy Ramirez, Jorge Gutiérrez-Cuevas, Arturo Santos-Garcia, Juan Armendariz-Borunda

**Affiliations:** 1 Department of Molecular Biology and Genomics, Institute for Molecular Biology in Medicine and Gene Therapy, Health Sciences University Center, University of Guadalajara, Guadalajara, Jalisco, Mexico; 2 School of Medicine and Health Sciences, Tecnologico de Monterrey, Monterrey, Mexico; 3 Department of Biomedical Sciences, Tonala University Center, University of Guadalajara, Tonala, Jalisco, Mexico; University of Navarra School of Medicine and Center for Applied Medical Research (CIMA), SPAIN

## Abstract

Adenoviral vector AdhMMP8 (human Metalloproteinase-8 cDNA) administration has been proven beneficial in various experimental models of liver injury improving liver function and decreasing fibrosis. In this study, we evaluated the potential therapeutic AdhMMP8 effect in a chronic kidney damage experimental model. Chronic injury was induced by orogastric adenine administration (100mg/kg/day) to Wistar rats for 4 weeks. AdhMMP8 (3x10^11^vp/kg) was administrated in renal vein during an induced-ligation-ischemic period to facilitate kidney transduction causing no-additional kidney injury as determined by histology and serum creatinine. Animals were sacrificed at 7- and 14-days post-Ad injection. Fibrosis, histopathological features, serum creatinine (sCr), BUN, and renal mRNA expression of αSMA, Col-1α, TGF-β1, CTGF, BMP7, IL-1, TNFα, VEGF and PAX2 were analyzed. Interestingly, AdhMMP8 administration resulted in cognate human MMP8 protein detection in both kidneys, whereas hMMP8 mRNA was detected only in the left kidney. AdhMMP8 significantly reduced kidney tubule-interstitial fibrosis and glomerulosclerosis. Also, tubular atrophy and interstitial inflammation were clearly decreased rendering improved histopathology, and down regulation of profibrogenic genes expression. Functionally, sCr and BUN were positively modified. The results showed that AdhMMP8 decreased renal fibrosis, suggesting that MMP8 could be a possible therapeutic candidate for kidney fibrosis treatment.

## Introduction

The 2012 Kidney Disease Improving Global Outcomes (KDIGO) defines CKD as kidney damage or a glomerular filtration rate (GFR) <60 mL/min/1.73 m^2^ for more than 3 months [1]. The guidelines define kidney damage as either functional abnormalities of the kidneys (such as proteinuria, electrolyte abnormalities or albuminuria, or abnormalities of the urinary sediment, such as dysmorphic red cells) or structural abnormalities as noted on imaging studies or histology, as well as, history of kidney transplantation [1] and is an increasing public health issue. Complications include increased all-cause and cardiovascular mortality, kidney-disease progression, acute kidney injury, cognitive decline, anaemia, mineral and bone disorders, and fractures. Worldwide, diabetes mellitus is the most common cause of chronic kidney disease, but in some regions, other causes, such as herbal and environmental toxins, are more common. CKD is considered an irreversible disorder which eventually progresses to fibrosis and renal failure; dialysis and ultimately kidney transplant are the only available treatments. Prevalence is estimated to be 8–16% worldwide. Renal fibrosis is the common final result of all CKD and it is a reliable prognosis marker of renal failure [2, 3]. International reports indicate that the prevalence of kidney fibrosis has increased around the world. As a result, CKD has become a major health problem causing a huge socio-economic burden to health systems [4, 5].

Despite the magnitude of this worldwide health problem, current renal fibrosis therapies are limited and often ineffective. In this context, conventional therapies for fibrosis in different organs have been focused in the inhibition of collagen synthesis or increasing metalloproteinases (MMPs) activity, as responsible for extracellular matrix (ECM) degradation. MMPs participate in the maintenance of the ECM protein scaffolds surrounding endothelium and are involved in kidney fibrosis and chronic remodeling, diabetic nephropathy, polycystic kidney disease and glomerulonephritis [6]. MMP8 (metalloproteinase 8) cleaves collagen I into 3/4 and 1/4 fragments becoming available for further degradation by gelatinases. MMP8 is a collagenase expressed by neutrophils and other cell types and is involved in homeostasis of collagens type I, II, III, VII and X along with other cytoskeletal proteins. MMP8 is stored as pro-enzyme in intracellular granules, and exocytosis is stimulated into the inflammation site by external signals. Latent form of MMP8 is activated extracellularly by other proteases and molecules from oxidative stress [7]. Recently, Basu and collaborators demonstrated a persistent histopathologic and functional injury and worsened health in MMP8-null mice subjected to acute kidney injury (AKI) suggesting MMP8 expression and activity seems to be directly proportional with recovery [8].

The aims of CKD treatments focus on controlling CKD progression and prevention of cardiovascular diseases, the principal cause of morbidity and mortality in the CKD population. Regenerative nephrology is emerging based on the idea that kidney regeneration could be achieved with the use of growth factors and morphogens, or multipotent stem cells that could be differentiated to restore and regenerate the chronically injured kidney. Both concepts require either recreation of a growth factor environment within the kidney to facilitate renal regeneration, or generation of renal type-specific progenitor cells *in vitro* to repopulate the kidney [6, 9].

On the other hand, gene therapy has experienced a refreshing resurgence, which undoubtedly will have an impact on therapeutic strategies for a number of chronic degenerative diseases. Nonetheless, no effective therapy has been devised for renal fibrosis [4, 10] in part due to the failure to consistently deliver transgenes to target cells throughout the kidney in a harmlessly approach. Gene therapy consists in the delivery of therapeutic genes to a target organ through different type of vectors, where viral vectors are mostly used. Our research group has demonstrated the therapeutic effect of different human proteins like urokinase Plasminogen Activator (uPA) [11, 12], ΔTGFbRII [13] (TGFβ1 truncated receptor) and MMP8 [10, 14, 15] coded by cognate cDNAs resulting in significantly reduced fibrotic tissue in experimental cirrhosis models. Thus, the aim of this study was to expand our knowledge in other fibrotic organs, and evaluate the therapeutic effect on kidney fibrotic tissue via administration of a recombinant human MMP8 cDNA cloned in an adenoviral vector in an adenine-induced CKD experimental model.

## Materials and methods

### Production of adenoviral vectors

Recombinant adenovirus serotype 5 carrying human *MMP8*cDNA (AdhMMP8) or the reporter gene *GFP* (AdGFP) were produced and amplified in our laboratory in cultured HEK-293 cells, using DMEM (Invitrogen, Grand Island, NY) supplemented with 10% fetal bovine serum (Invitrogen, Grand Island, NY), 37°C and 5% CO2 atmosphere. Cells were collected after 48h of adenoviral transduction and disrupted using three freeze/thawing cycles. Cellular debris was removed by centrifugation and supernatant containing rAd was concentrated by two ultracentrifugation rounds at 141,000 RCF in a CsCl density gradient. AdhMMP8 and AdGFP particles per milliliter were determined by optical densitometry (OD), and infection units quantification was determined by end-point titration [16].

### Adenoviral vector bio-distribution

To evaluate adenoviral bio-distribution and corroborate transgene expression, GFP expression was monitored in liver, lung, spleen and kidney. 3x10^11^vp/kg of AdGFP was administered in the left renal vein by retrograde venous hydrodynamic injection in healthy rats; after either 5 or 15 minutes of ischemia by clamping both left renal vein and artery. Kidney reperfusion was permitted immediately thereafter. Animals were sacrificed 48h after AdGFP transduction and reporter gene expression was quantified in at least 20 stereoscopy-zoomed photographs. Percentage of GFP expression was calculated using Image J^®^ program.

### Animal model

Thirty male Wistar rats (~250g) were orogastrically intoxicated with adenine (100mg/kg/day) (Sigma-Aldrich, St Louis, MO, USA) for 4 weeks, a modification of the Yokozawa and col. [17, 18] animal model for chronic renal failure. Ten animals were used as healthy controls and orogastrically administered with vehicle within the same period of time. Animals were divided in 4 groups: Healthy control group (n = 10) without adenine administration. Adenine control group (ADENINE; n = 10); adenine intoxicated plus vehicle in renal injection. MMP8 treated group (ADE + MMP8; n = 10) was administrated with 3x10^11^vp/kg of AdhMMP8 via left kidney vein. Also, ADE + GFP group (n = 10), as transgene control group; that was injected in the left kidney vein with AdGFP (3x10^11^vp/kg). A subset of half of the animals of each group, were sacrificed at 7 and 14 days after adenovirus administration. At sacrifice, blood was collected through cardiac-punction and both kidneys were removed and sampled.

Animals were obtained from the animal facility at Health Sciences University Center of University of Guadalajara and housed in a maximum of 4 animals per cage. Rats received care according to the Mexican Official Norm NOM-062-ZOO-1999 and proper guidelines of University of Guadalajara using 12h light/dark cycles. Rats were fed *ad libitum* with free access to plain water. The protocol was approved by the Research and Ethical Committees of CUCS, University of Guadalajara (approval number CEI/282/2016).

### Adenovirus administered by retrograde venous hydrodynamic injection

We used hydrodynamics-based transfection to improve the access and spreading of viral vectors in renal cells and structures. Hydrodynamic delivery (HD) involves a pressurized injection of a large volume of solution into a vasculature; this process increases the permeability of the capillary endothelium and epithelial junctions, and generates transient pores in cell membranes facilitating the internalization of RNA, DNA or viral particles. For adenovirus administration animals were anesthetized with tiletamine/zolazepam (100mg/20mg/kg), asepsis was performed on the abdomen and midline incision was made to expose the renal hilum. Left kidney vein was secured with micro vascular clamps and 3x10^11^vp/kg of AdhMMP8 or AdGFP were injected by retrograde way. Clamp was maintained in vein and artery for either 5 or 15 minutes, and after these times renal blood flow was restored and incision was closed and animals were allowed to recuperate.

### Renal function tests determination

Blood was collected and serum was obtained. Serum levels of creatinine and blood urea nitrogen (BUN) were measured in an automated Vitros DT 60 equipment (Johnson and Johnson, New Jersey, NY).

### Kidney histological analysis

Kidneys were sliced in 2–3 mm segments and fixed by immersion in a 4% paraformaldehyde solution, dehydrated, and embedded in paraffin. Sections 5 μm thick were stained with Hematoxylin& Eosin (H&E) and Masson’s trichrome to assess tissue damage and inflammation, as well as the degree of renal fibrosis. Kidney fibrosis was quantified in 20 Masson’s trichrome stained tissue microphotographs using a computer-assisted image analyzer (Image-ProPlus 6.0, Media Cybernetics, Inc., Bethesda, MD) while inflammatory cell infiltration was quantified in H&E stained tissue. In addition, interstitial fibrosis, tubular atrophy, interstitial inflammation and mesangial matrix increase was estimated by a pathologist blind to the study using Banff Classification of Kidney Allograft Pathology [18, 19].

### Gene expression analysis

Total RNA was isolated according to Chomczynski and Sacchi modified method [20]. Briefly, kidney tissue was homogenized in the presence of Trizol reagent (Invitrogen, Carlsbad, CA). Chloroform was added and the aqueous phase was isolated. RNA was precipitated with isopropanol. RNA quantity and quality were determined in NanoDrop equipment (Thermo Scientific, USA). For retrotranscription, 2 μg of total RNA were used with 240 ng Oligo dT, 0.5 mM dNTPs mix, 10 mM DTT, 2 U of RNAse inhibitor and 200 U MMLV (Invitrogen, Carlsbad, CA). After the RT cycle, samples were stored at -70°C until use. Two μL of cDNA were subjected to Real-Time PCR using the LightCycler (Roche Life Sciences, Pleasanton, CA, USA) under the following conditions 2 min/50°C, 10 min/95°C, and 45 cycles of 15 sec/95°C and 1 min/60°C. Specific primers and probes designed to align with Collagen 1α (*Col-1*α), α -smooth muscle actin (α*-SMA*), transforming growth factor β1 (*TGF*-β1), connective tissue growth factor (*CTGF*), vascular endothelial growth factor (*VEGF*), bone marrow protein-7 (*BMP*-7), tumor necrosis factor-α (*TNF*-α), interleukin-1β (*IL-*1β), paired Box 2 (*Pax2)*, human *MMP8* and *GAPDH (*glyceraldehyde-3-Phosphate Dehydrogenase), as housekeeping gene, were acquired from Applied Biosystems (Hammonton, NJ, United States). Gene amplification was normalized against GAPDH expression with the healthy group as internal calibrator. Relative quantification by the 2^-ΔΔCT^ method was performed.

### ELISA assay for human MMP8

Proteins were extracted from 100–150 mg of kidney tissue minced in a buffer solution (pH 7.4) containing 65 mmol/L Tris, 310 mmol/L KCl, 1% Nonidet P40, 0.1% SDS, sodium orthovanadate 200 mmol/L, 1 mol/L NaF and 1X complete protease inhibitor cocktail tablets (Roche Diagnostics, Indianapolis, IN). After centrifugation at 21000 rcf/4°C for 1 hour, supernatant was isolated and stored at -80°C until required. Total protein quantification was performed using a Bradford assay [21]. Human MMP8 was quantified using a human total MMP8 ELISA kit from R&D Systems (Minneapolis, United States) according to the manufacturer instructions.

### Statistical analysis

Data are represented as mean ± SD. Each of the 4 groups at each point of the sacrifice were compared with Kruskal-Wallis test followed by Mann-Whitney test. Differences were considered to be statistically different at p<0.05. Analysis was run with SPSS 18.0.

### Methods statement

All methods were carried out in accordance with relevant guidelines and regulations.

## Results

### Macroscopic analysis of kidney tissue

**Fig 1:**
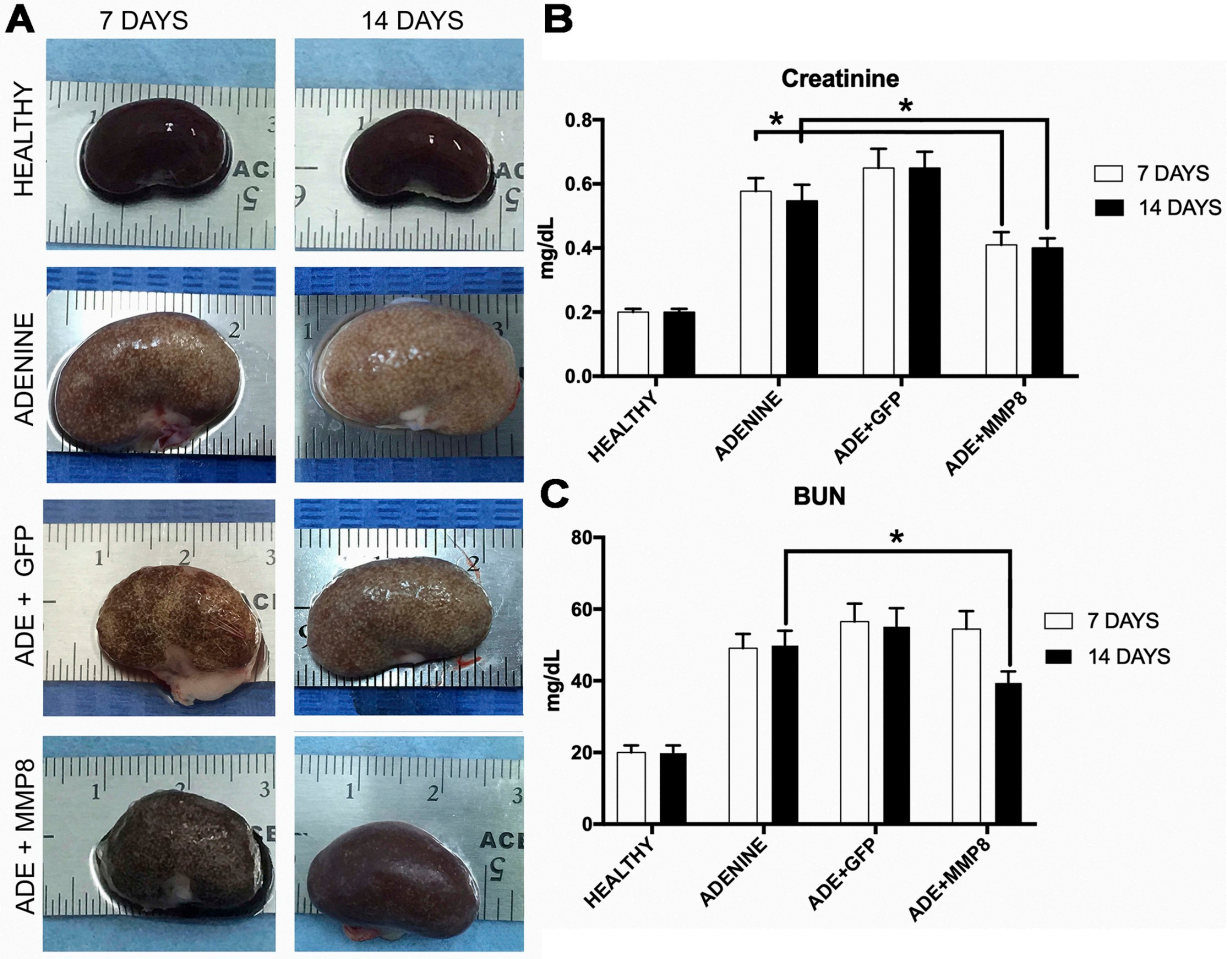
Macroscopic appearance of the kidneys and renal functional tests. **A)** Image shows representative kidneys of healthy and adenine controls, irrelevant AdGFP and AdhMMP8 treated animals at day 7 and 14 after vector administration. Adenine crystal deposition can be observed in the intoxication model. A notable diminution in adenine deposition can be observed in AdMMP8 treated animals. **B)** Blood Ureic Nitrogen (BUN) serum levels and **C)** serum creatinine levels at 7 and 14 days after intervention. * denotes a p<0.05.

All animal groups were sacrificed 7 and 14 days after adenovirus administration, and the kidneys were obtained. Macroscopic observations showed enlargement, altered color and texture of kidneys in adenine group when compared to healthy group (Fig 1). Additionally, kidneys from rats injured with adenine presented crystal precipitations and nephrolithiasis. Noteworthy, at day 14 AdhMMP8 injected animals showed macroscopically a well-defined improvement in appearance which was not observed in the AdGFP control group (Fig 1A).

### AdhMMP8 administration improved renal function

We evaluated the effect of AdhMMP8 therapy on renal function indicators. Serum levels of creatinine and BUN increased significantly due to adenine intoxication (p>0.05, Fig 1B and 1C). In AdhMMP8 groups, serum creatinine levels diminished at day 7 and persisted decreased until day 14 compared to both adenine and AdGFP-injected animals (Fig 1B). BUN levels decreased significantly 14 days after AdhMMP8 treatment (p>0.05; Fig 1C). As expected, creatinine and BUN remained increased in irrelevant AdGFP group.

### Adenoviral biodistribution

To determine bio-distribution of adenoviral particles, animals were administrated with 3x10^11^vp/kg of AdGFP via left renal vein and sacrificed 72h later. Renal ischemia was sustained for 5 or 15 minutes due to renal vein and artery clamping.

Kidneys, lungs, liver and spleen were obtained and observed under epifluorescence stereoscopy to evaluate the green fluorescent protein (GFP) expression. Five macroscopic photographs of each organ were analyzed and transduction percentage was determined according to GFP expression. GFP expression was higher in animals exposed 15 minutes to ischemia compared with 5 minutes (S1A Fig). After 5 minutes of ischemia, GFP expression was 42.5± 2.5% in left kidney, 25.5± 0.6% in liver, and 5.3± 0.2% in spleen. Contrasting with these previous data, after 15 minutes of ischemia, GFP expression was 72.5 ± 5% in left kidney, 7.5 ± 5% in liver, and 6.7 ± 0.7% in spleen. No fluorescence was detected in the right kidney at any time (S1B Fig). Expression of reporter gene in a particular renal cell type was not determined, since our aim was to evaluate total renal expression regardless of the cell type expressing the transgene. These results indicate that most of the adenoviral vector particles remained in the injected renal tissue (S1B Fig).

### AdhMMP8 therapy diminished renal fibrosis and inflammatory cellular infiltration

**Fig 2:**
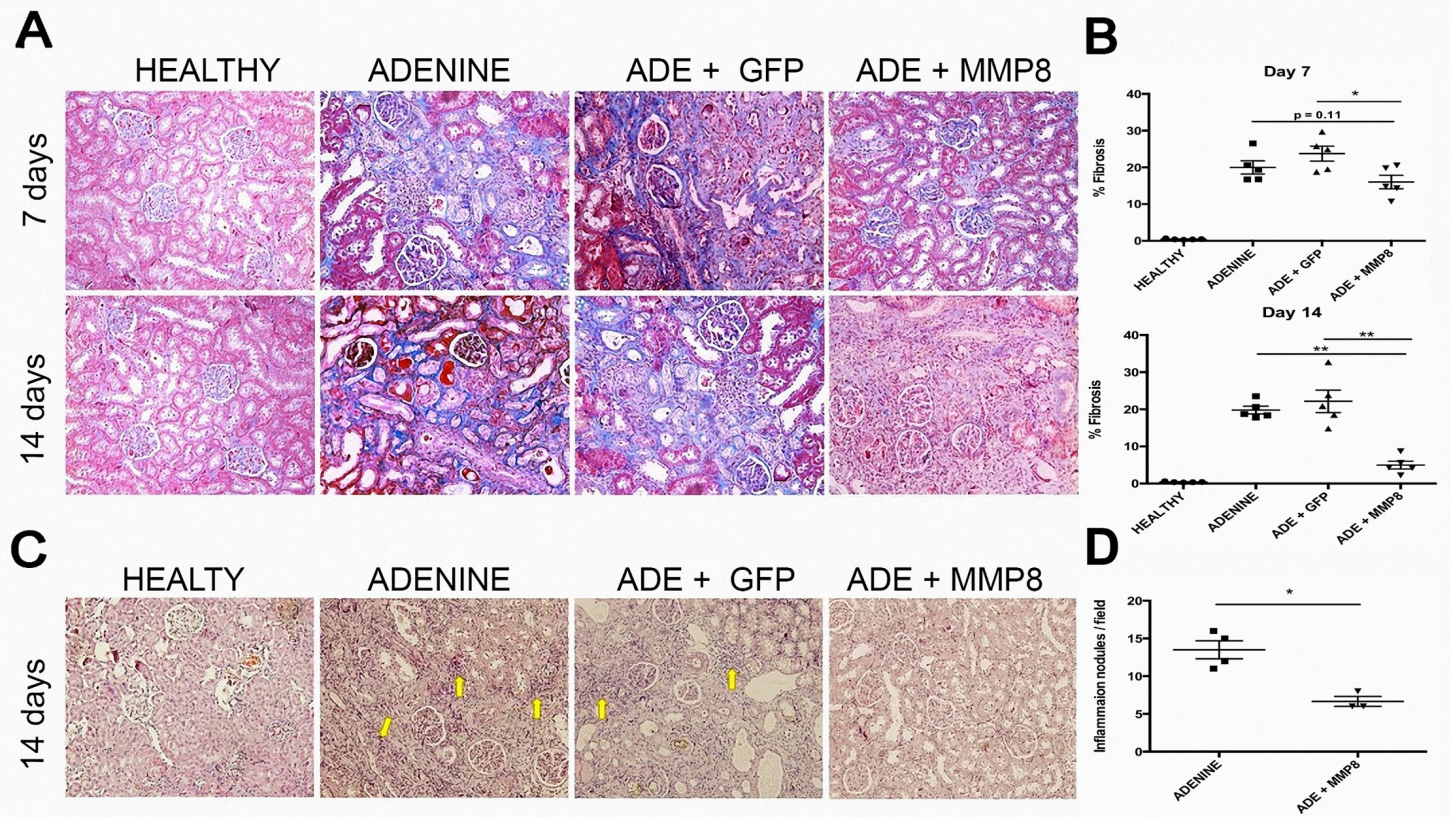
Assessment of kidney fibrosis and inflammation. **A)** Masson staining showed morphological tissue alteration in Adenine group with established fibrosis. Animals treated with AdhMMP8 vector showed a significant marked reduction of fibrotic tissue at both cohort times. **B)** Graphs showed percentage of fibrosis in renal tissue according to software analysis of representative images. Fibrosis is notably reduced in AdMMP8 groups, especially at day 14 after adenoviral administration. ** p<0.01, * p<0.05. **C)** Adenine group H&E staining showed granulomas formation and inflammatory cell infiltrates. Also, zones of tubular atrophy with interstice expansion as a consequence of fibrosis and mononuclear inflammatory cell nodules were noted. A decrease of inflammatory cells, fibrotic tissue and inflammatory nodules (yellow arrows), was clearly seen 14 days after AdhMMP8 administration. Likewise, treated animals revealed a decrease in interstitial fibrosis and improvement in tissue architecture and tubular structure. **D)** Graph showed the quantification of the renal inflammatory cell foci. *p<0.05.

Histopathological findings obtained for renal tissue of the different groups are shown in Fig 2A and 2B. According to the evaluation of the pathologist blind to the study and based on the Banff classification of renal allograft pathology, renal tissue presented a normal architecture in healthy rats. On the other hand, in adenine and AdGFP groups, an exacerbated infiltration of inflammatory cells can be seen, along with granulomas containing crystals and inflammatory cells. The principal renal injury is observed at the interstitial tubular level with atrophy zones, acute tubular necrosis, tubulitis and adenine crystals in the inner part of several tubules. Also, an interstice expansion as a consequence of fibrosis and mononuclear inflammatory cell nodules (lymphocytes and plasmatic cells) was observed; as well as multinucleated giant cells like strange bodies around the adenine crystals and some neutrophils. At the glomerular level, marginal changes can be observed with <25% glomeruli affected without hypercellularity.

In AdhMMP8 treated animals, fibrotic tissue and inflammatory nodules significantly declined with less interstitial space and few inflammatory cells. The persistent nodules are smaller with a minor number of inflammatory cells. Also, a slight quantity of multinucleated giant cells was observed (Fig 2A and 2C). The reduction of renal inflammatory cell infiltration was quantified using image analysis software in representative photographs (Fig 2D).

**Fig 3:**
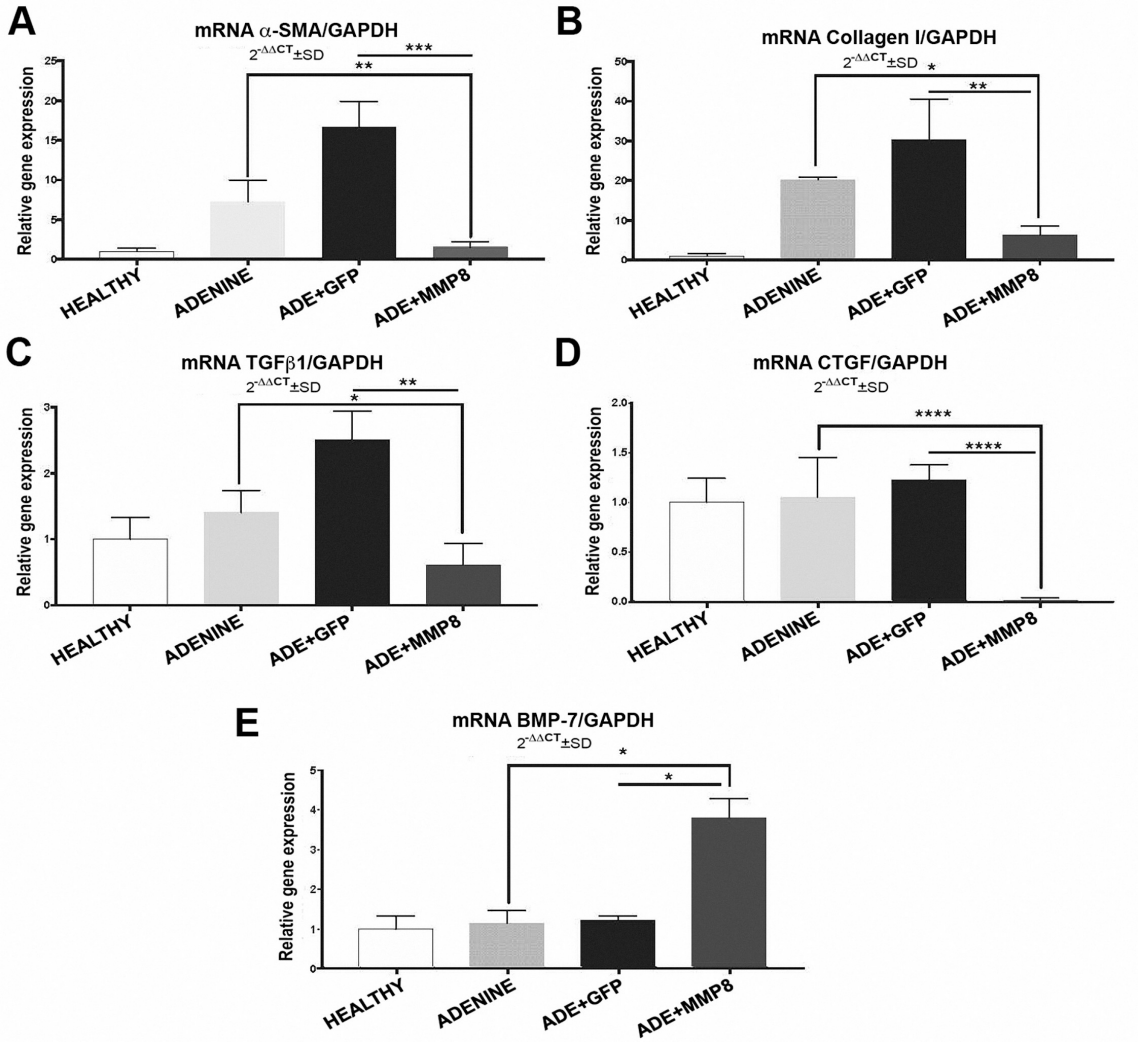
mRNA levels of profibrogenic and anti-fibrogenic genes. **A-D)** Fold change in gene expression indicates that adhMMP8 treatment clearly decreased αSMA, Col1A1, TGFβ1 and CTGF mRNA levels; and increases **E)** BMP7 mRNA values compared to controls. (*p<0.05, **p<0.01, ****p<0.0001).

The percentage of renal fibrosis was measured in Masson stained kidney sections using a computer-assisted morphometric analysis (Fig 2B). Normal kidney architecture was observed in healthy control animals presenting 0.44 ± 0.15% of fibrosis. Adenine-group showed excess of extracellular matrix with disruption of parenchymal architecture with 19.9± 4.1% of fibrosis. AdhMMP8 treated animals showed a reduction in fibrosis around 20% at day 7 post-treatment and 75% 14 days after AdhMMP8 administration compared to Adenine-group. We believe this result can be due to the fact that adenoviral transduction reached its peak levels approximately at day 12 according to previous data [12]. Irrelevant-transgene (AdGFP) administration did not show any beneficial effect in fibrosis (Fig 3A). Importantly, adenine model does not show spontaneous reversion. This can be verified, since fibrosis values between day 7 and 14 are not statistically different in adenine group, nor even in AdGFP group.

According to Banff classification summarized in Table 1, adenine group was situated in score 2–3 (45.6±22.4%) for interstitial infiltrate, whereas AdhMMP8 group scored 1–2 (14.25±1.7%), showing a marked decrease in inflammatory cell infiltrate (p<0.01). Interstitial fibrosis in adenine group scored 2–3 with elevated fibrosis percentage in the cortical area (19.9±4.0%). A significant decrease (p<0.01) was observed in AdhMMP8 group with samples scoring 0–1 with very low percentages (5.1±1.4%) of fibrosis. Tubular atrophy showed a moderate improvement when AdhMMP8 was administered scoring 0–1 (20.67±7.2%) compared to a score of 2 (37.8±7.8%) in adenine group. AdGFP group did not show differences when compared with adenine group. Histological indicators for renal damage based on the Banff classification are reviewed in Table 1.

**Table 1:** Banff classification.

	Histological Indicators of Renal Damage		
	Left kidney		
Group	Interstitial Fibrosis	Tubular Atrophy	Interstitial Inflammation
**HEALTHY**	**0 **(0.44% ± 0.15)	**0 **(0.8% ± 0.6)	**0 **(1.6% ± 0.8)
**ADENINE**	**2.2 **(19.9% ± 4.0)	**2.2 **(37.8% ± 7.8)	**2 **(45.6% ± 22.4)
**ADE+AdGFP**	**2.2 **(21.5% ± 5.1)	**2.2 **(36.7% ± 6.4)	**2 **(45.3% ± 19.2)
**ADE+AdhMMP8**	**1.6 **(5.1% ±1.4)**	**1.6 **(20.67% ± 17.2)	**1 **(14.25% ± 1.7)**

(**p<0.01) As compared to adenine group of rats.

### Expression of human MMP8 gene and protein in animals treated with AdhMMP8

Human MMP8 protein was quantified using specific ELISA assay that excludes rodent MMP8, to corroborate transduction of AdhMMP8 into the kidneys of adenine-intoxicated animals. Thus, even when some neutrophils infiltration could be taken place as a result of the injury; this event should not interfere with the obtained hMMP8 levels. Also, rat MMP8 is not expected to be modified by the treatment. hMMP8 levels in left kidney homogenates was 9.057±1.96pg/μg of total protein at day 7 and 6.38±2.71pg/μg of total protein at day 14. Surprisingly, we also detected hMMP8 in right kidney homogenates where AdhMMP8 was not administrated. Values were 10.034±1.92 pg hMMP8/μg of total protein at day 7 and 2.389±0.52 pg/μg at day 14 (S2A Fig). Nonetheless, and as anticipated, human MMP8 mRNA was detected only in the left kidney where AdhMMP8 viral particles were primarily administered (S2B Fig).

Since MMP8 expression was found in the contralateral kidney; histological analysis (S3 Fig) and histological indicators for renal damage based on the Banff classification were reviewed and included in S1 Table.

### Expression of profibrogenic genes decreased by Ad-hMMP8 therapy

Fourteen days after adenoviral administration, expression of profibrogenic genes like α-SMA (1.55±0.71 vs 7.27±2.7; p<0.01), Col-1α (6.21±2.3 vs 20.25±0.64, p<0.05), TGFβ1 (0.61±0.33 vs 1.41±0.33, p<0.05) and CTGF (1.05±0.39 vs 0.1±0.03, p<0.01) was significantly down-regulated in AdhMMP8 treated animals compared with their non-treated counterparts (Fig 3). Importantly, all these genes exhibited a dramatic reduction when compared with irrelevant AdGFP administered animals (p<0.01). Noteworthy, no statistic significant differences were detected between AdGFP group and control adenine group. The anti-fibrotic gene BMP7 was significantly up-regulated (3.8±0.48 fold-change) in the group treated with AdhMMP8 compared with adenine control group (1.15±0.32 fold-change, p<0.05) in Fig 3E.

**Fig 4:**
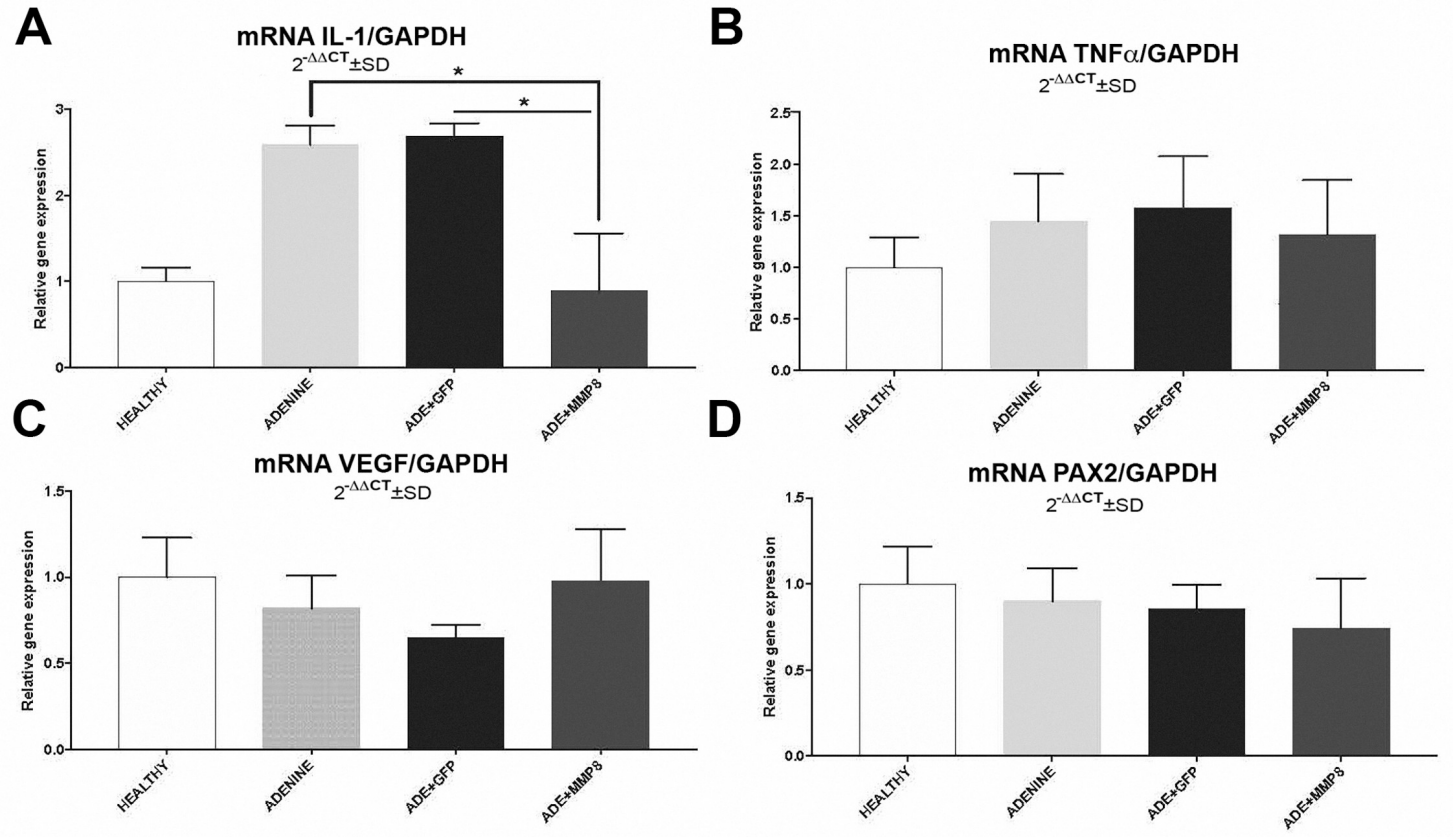
Gene expression of proinflammatory and renal tissue-remodeling genes. **A)** IL-1β gene expression profile showed diminution after adhMMP8 treatment (*p<0.05). **B–D)** TNFα, VEGF and PAX2 mRNA levels did not showed significant modifications compared to controls.

In the case of inflammation related molecules, mRNA level of IL-1β (Fig 4A) significantly decreased after 14 days of AdhMMP8 administration (0.89±0.97 vs 2.59±0.22, p<0.05); while TNFα showed no statistical difference compared to adenine controls (1.32±0.53 vs 1.45±0.46, Fig 4B). No statistically significant differences between adenine and AdGFP control group were observed for IL1β, neither TNFα. Renal tissue remodeling genes like VEGF and PAX2 (Fig 4D), did not display significant changes with MMP8 therapy compared to adenine control group; though VEGF showed a tendency to up-regulation (Fig 4C).

## Discussion

Several publications provide a comprehensive overview of recent progresses using viral vectors for direct gene transfer to the kidney. Different strategies have been developed to demonstrate viral vector transduction in all types of cells within the kidney, including the glomeruli, tubular epithelial cells in the cortex and medulla, and interstitial cells [22, 23]. Important to state, is the fact that in this work, total renal expression of hMMP8 was the primary aim of AdMMP8 administration, regardless of the type of a specific protein-expressing cell.

Here we used adenoviral vectors to achieve a proof of concept, implementing adenovector delivery to the kidney via retrograde renal vein injection. In this study we show that AdhMMP8 administration promotes amelioration of kidney fibrosis, reducing the inflammatory cellular infiltration and down-regulating of profibrogenic and proinflammatory genes, such as, TGF-β1, Col1α, α-SMA, CTGF and IL1β. In our previous reports using this human cDNA for MMP8 in experimental liver cirrhosis, we established a significant reduction of pro-fibrogenic genes expression, an important reduction of fibrosis index and hepatic stellate cells proliferation [10]. Outstandingly, adenoviral-delivered human-MMP8 expression and most importantly: activation inside rat tissue, was already proved [14]. These data support the reproducibility and the feasibility of fibrosis treatment with this genomic approach for both tissues: liver and kidney.

Adenovirus innate liver tropism has been widely described [24, 25], to overcome this limitation; viral particle modification and innovations in the administration strategies have been development to target renal tissue [26, 27]. Administration route had proved to be an important factor regardless of the gene vector being considered. In this study, administration strategy is based in hydrodynamic injection and a clamping-induced ischemia period to increase the blood supply into the kidney, increasing the possibility of renal transduction and reducing rAd liver transduction and viral particles trapping in lungs and spleen. AdGFP administration via retrograde injection into the renal vein showed best transduction with 15 minutes of ischemia, time elected for the AdhMMP8 therapy administration. MMP8 mRNA detection clearly shows that MMP8 is transduced only in the injected kidney, data corroborated by GFP biodistribution that showed only GFP expression in the left kidney. Despite that liver GFP transduction was detected, we consider main amounts of MMP8 protein detected in right kidney came from left kidney cells. Liver cells positive for GFP were limited to the organ surface, suggesting a possible liquid leaking coming from the left kidney that only reach the hepatic exterior.

MMPs have both inhibitory and stimulatory roles in fibrosis [28], the role of MMPs in renal diseases is controversial. Also, there is a lack of information of MMP8 as a therapeutic molecule and even more in renal fibrosis disease. However, Basu and col. [8] observed MMP8 differential expression in patients with severe sepsis-associated acute kidney injury (AKI). In an experimental model of AKI they demonstrated MMP8 involvement in purely ischemic AKI using MMP8 null mice, which presented a persistent histopathologic and functional injury and worsened health versus wild-type animals. Thus, these authors suggested that MMP8 is involved in kidney injury restoration. To our knowledge, there are no additional studies evaluating MMP8 effect specifically on kidney fibrosis; or in adenine model where MMP1 is the metalloproteinase that has been reported to increase [29].

We showed the results of 2 different cohorts time periods at 7 and 14 days after AdhMMP8 administration. We observed a significant reduction (p>0.01) on fibrosis at day 14 representing a fibrosis reversion of 75% compared to adenine group, result even higher than the one achieved in liver cirrhosis models (30%-60%) [10, 14, 30]. This difference could be explained by tissue architecture, organ size and cellular location of profibrogenic cells in the kidney, (*i*.*e*., kidney pericytes and perivascular fibroblasts) alongside with adenovirus particles capability to reach and transduce specific tissue zones. Upon delivery of the adenoviral via administration, the tissue zone transduced differs. Delivery of Ad vector into the renal artery of adult rats resulted in gene transduction into proximal tubular cells, but not into the glomeruli [31]. In another study, the same via resulted in a significant gene transfer to cells of the renal cortex [32]. In our study, GFP expression seems to be present in most of the renal tissue, including cortex and medulla; which could lead to a significant transgene expression. However, it is important to further evaluate longer ischemic times and other routes of administration to achieve maximum renal expression of the therapeutic gene hMMP8.

After day 14 of AdhMMP8 injection, the histopathologic analysis by a pathologist unsighted to the study, showed an important tissue improvement, represented by decreased inflammatory nodules, less interstitial space and fewer number of inflammatory cells. The tissue presented persistent, but smaller, inflammation nodules compared to the adenine control group and a smaller number of multinucleated giant cells. This regeneration was observed mainly in tubular cells in a progressive manner throughout the treatment which is coincident with previous reports in the literature where kidney regeneration is possible only in tubular cells [33, 34]. These findings where observed also in a minor degree in the right kidney (S3 Fig). Right kidney tissue presented most of the glomeruli in viable conditions without double contour of the basal membrane, necrosis was not observed, and all capillary were permeable. According to the Banff classification the focal tubulitis was T1 (S1 Table). Possible explanations are that, since MMP8 protein was detected in lesser amounts in the right kidney, the beneficial effect was also minor; but enough to be histologically noticeable. Also, since right kidney was not subjected to ischemia, this can positively influence the histological analysis. Several researchers have proposed that ECM remodeling can be due to the release of growth factors produced by the degradation of matrix proteins by metalloproteinases [35–37]. In this context we observed regeneration of tubular cells after AdhMMP8 treatment which matches with what is proposed by these researchers. However, more experiments are required to corroborate these results in the light that specific genes like VEGF and PAX involved in renal tissue remodeling were found not meaningfully increased.

Functionally, animals treated with AdhMMP8 showed a significant improvement on serum BUN and creatinine levels which offers an acceptable outcome and warrants further research.

On the other hand, recombinant adeno-associated virus (AAV) is a non-integrating, non-enveloped, and replication deficient parvovirus that has emerged as a tool for gene delivery. Recently Ikeda and col. [38] evaluated the transduction profiles of six pseudotyped AAV vectors expressing reporter’s genes in mouse kidney and human organoids. Of the six vectors, a synthetic AAV (Anc80) showed specific and high-efficiency transduction of kidney mesenchymal cells, including pericytes, fibroblasts and mesangial cells. Also, they validated the use of Anc80 for gene transfer by knocking out Gli2 from kidney mesenchyme using Anc-80 Cre, confirming an antifibrotic effect after chronic obstruction. Adenovirus and AAVs have different characteristics with different advantages and disadvantages as a gene transfer vectors. As far as we are concerned, we believe the gene therapy strategy shown here achieved efficient kidney transduction resulting in antifibrotic therapy using hMMP8 as a therapeutic protein.

The results of the present work represent the proof of concept for a new possible renal fibrosis therapy approach in humans, though additional research is needed before applying it into potential clinical settings.

## Supporting information

S1 Fig Adenoviral vector biodistribution. **A)** Photographs of the reporter gene AdGFP expression in the left kidney, right kidney, spleen and liver after 5 and 15 minutes of administration via retrograde renal vein injections. **B)** Graph shows percentage of GFP expression according to image analysis mice tissues at 5 and 15 minutes after AdGFP administration.
(TIF)

S2 Fig Human MMP8 protein detection in mouse renal tissue. **A)** Total hMMP8 protein quantification is presented separately in left and right kidney homogenates at day 7 and 14 after adenoviral administration. **B)** mRNA detection of transduced human MMP8 cDNA in mouse renal samples clearly showed that it is only present in left kidney tissue.
(TIF)

S3 Fig Histological analysis in right kidney. **A)** Panoramic view of the renal cortex. Adenine crystal deposits in the tubules can be observed and, as response to chronic damaged, tubules dilatation is noticed (HE staining, 10X). **B)** Tubules with frayed cytoplasm. According to the Banff classification, the focal tubulitis was T2, tissue showed 35% of tubular atrophy and 32.5% of the tissue presented mononuclear inflammatory cells foci (HE staining, 40X). **C)** Masson staining showed approximately 35% of interstitial fibrosis (10X). **D)** Panoramic view of the renal cortex; more preserved tissue histology can be observed. The grade of glomerulitis was G1 (HE staining, 10X). **E)** Renal tubules presented dilatation and frayed cytoplasm. According to Banff classification, focal tubulitis was T1. Tissue showed 15% of tubular atrophy (grade 1: ≤25%) (HE staining, 40X). **F)** Masson staining showed some areas of interstitial fibrosis. Thus, in the 12.5% of the renal cortex, the interstice space was enlarged in consequence (10X).
(TIF)

S1 Table Banff classification. (DOCX)

## References

[1] Kidney Disease: Improving Global Outcomes (KDIGO) CKD Work Group. KDIGO 2012 Clinical Practice Guide-line for the Evaluation and Management of Chronic Kidney Disease. Kidney Int Suppl. 2013;3:1–150.

[2] Tampe D, Zeisberg M. Potential approaches to reverse or repair renal fibrosis. Nat Rev Nephrol. 2014;10(4): 226–237. 10.1038/nrneph.2014.14 24514753

[3] Lan A., Du J. Potential role of Akt signaling in chronic kidney disease. Nephrol Dial Transplant. 2015;30(3): 385–394. 10.1093/ndt/gfu196 24891436

[4] Méndez-Durán A, Méndez-Bueno JF, Tapia-Yáñez T, Muñoz Montes A, Aguilar-Sánchez L. Epidemiología de la insuficiencia renal crónica en México. Diálisis y Trasplante. 2010;31(1): 7–11.

[5] Hewitson TD. Fibrosis in the kidney: is a problem shared a problem halved? Fibrogenesis Tissue Repair. 2012; 5(Suppl 1):S14 10.1186/1755-1536-5-S1-S14 23259697 PMC3368763

[6] Tan RJ, Liu Y. Matrix metalloproteinases in kidney homeostasis and diseases. Am J Physiol Renal Physiol. 2012; 302(11): F1351–F1361. 10.1152/ajprenal.00037.2012 22492945 PMC3774496

[7] Khokha R, Murthy A, Weiss A. Metalloproteinases and their natural inhibitors in inflammation and immunity. Nat Rev Immunol. 2013;13(9):649–665. 10.1038/nri3499 23969736

[8] Basu RK, Donaworth E, Siroky B, Devarajan P, Wong HR. Loss of matrix metalloproteinase-8 is associated with worsened recovery after ischemic kidney injury. Ren Fail. 2015; 37(3):469–475. 10.3109/0886022X.2014.996842 25578815 PMC4414708

[9] Corridon PR, Rhodes GJ, Leonard EC, Basile DP, Gattone 2nd VH, Bacallao RL, et al A method to facilitate and monitor expression of exogenous genes in the rat kidney using plasmid and viral vectors. Am J Physiol Renal Physiol. 2013; 304(9): F1217–F1229. 10.1152/ajprenal.00070.2013 23467422 PMC3651629

[10] Siller-López F, García-Bañuelos J, Hasty KA, Segura J, Ramos-Márquez M, Qoronfleh MW, et al Truncated active matrix metalloproteinase-8 gene expression in HepG2 cells is active against native type I collagen. J Hepatol. 2000; 33(5): 758–763. 10.1016/s0168-8278(00)80307-4 11097484

[11] Meza-Rios A, García-Benavides L, García-Bañuelos J, Salazar-Montes A, Armendáriz-Borunda J, Sandoval-Rodríguez A. Simultaneous administration of ADSCs-based therapy and gene therapy using Ad-huPA reduces experimental liver fibrosis. Plos One. 2016;11(12):e0166849 10.1371/journal.pone.0166849 27992438 PMC5161330

[12] Salgado S, Garcia J, Vera J, Siller F, Bueno M, Miranda M, et al Liver cirrhosis is reverted by urokinasetype plasminogen activator gene therapy. Mol Ther. 2000; 2(6): 545–551. 10.1006/mthe.2000.0210 11124055

[13] Marquez-Aguirre A, Sandoval-Rodriguez A, Gonzalez-Cuevas J, Bueno-Topete M, Navarro-Partida J, Arellano-Olivera I, et al Adenoviral delivery of dominant-negative transforming growth factor beta type II receptor up-regulates transcriptional repressor SKI-like oncogene, decreases matrix metalloproteinase 2 in hepatic stellate cell and prevents liver fibrosis in rats. J Gene Med. 2009;11(3): 207–219. 10.1002/jgm.1303 19189315

[14] Siller-Lopez F, Sandoval A, Salgado S, Salazar S, Bueno M, Garcia J, et al Treatment with human metalloproteinase-8 gene delivery ameliorates experimental rat liver cirrhosis. Gastroenterology. 2004;126(4):1122–1133. 10.1053/j.gastro.2003.12.045 15057751

[15] Gálvez-Gastélum FJ, Garcia-Bañuelos JJ, Beas-Zárate C, Segura-Flores A, González H, Chaparro-Huerta V, et al Combinatorial gene therapy induces regression of hepatic encephalopathy. Gene Ther. 2011;18(1): 88–94. 10.1038/gt.2010.107 20703313

[16] Armendáriz-Borunda J, Bastidas-Ramírez BE, Sandoval-Rodríguez A, González-Cuevas J, Gómez-Meda B, García-Bañuelos J. Production of first generation adenoviral vectors for preclinical protocols: amplification, purification and functional titration. J Biosci Bioeng. 2011;112(5): 415–421. 10.1016/j.jbiosc.2011.07.018 21856222

[17] Yokozawa T, Zheng PD, Oura H, Koizumi F. Animal model of adenine-induced chronic renal failure in rats. Nephron Exp Nephrol. 1986;44(3):230–234. 10.1159/000183992 3785486

[18] Rivera-Valdés J, García-bañuelos J, Salazar-Montes A, García-Benavides L, Dominguez-Rosales A, Armendariz-Borunda J, et al Human adipose derived stem cells regress fibrosis in a chronic renal fibrotic model induced by adenine. PLoS ONE. 2017;12(12): e0187907 10.1371/journal.pone.0187907 29281649 PMC5744925

[19] Racusen LC, Solez K, Colvin RB, Bonsib SM, Castro MC, Cavallo T, et al The Banff 97 working classification of renal allograft pathology. Kidney Int. 1999;55(1999):713–723. 10.1046/j.1523-1755.1999.00299.x 9987096

[20] Chomczynski P, Sacchi N. Single-step method of RNA isolation by acid guanidinium thiocyanate-phenol- chloroform extraction. Anal Biochem. 1987;162(1):156–159. 10.1006/abio.1987.9999 2440339

[21] Bradford MM. A rapid and sensitive method for the quantitation of microgram quantities of protein utilizing the principle of protein-dye binding. Anal Biochem. 1976;72(1–2): 248–254. 10.1006/abio.1976.9999 942051

[22] Davis L, Park F. Gene therapy research for kidney diseases. Physiol Genomics. 2019; 51(9):449–461. 10.1152/physiolgenomics.00052.2019 31398086

[23] Rubin JD, Nguyen TV, Allen KL, Ayasoufi K, Barry MA. Comparison of Gene Delivery to the Kidney by Adenovirus, Adeno-Associated Virus, and Lentiviral Vectors After Intravenous and Direct Kidney Injections. Hum Gene Ther. 2019;30(12):1559–1571. 10.1089/hum.2019.127 31637925 PMC6919283

[24] Nakamura T, Sato K, Hamada H. Reduction of natural adenovirus tropism to the liver by both ablation of fiber-coxsackievirus and adenovirus receptor interaction and use of replaceable short fiber. J Virol. 2003;77(4):2512–2521. 10.1128/jvi.77.4.2512-2521.2003 12551989 PMC141073

[25] Alemany R, Curiel DT. CAR-binding ablation does not change biodistribution and toxicity of adenoviral vectors. Gene Ther. 2001;8(17):1347–1353. 10.1038/sj.gt.3301515 11571572

[26] Nahman NS, Sferra TJ, Kronenberger J, Urban KE, Troike AE, Johnson A, et al Microsphere-adenoviral complexes target and transduce the glomerulus in vivo. Kidney Int. 2000;58(4):1500–1510. 10.1046/j.1523-1755.2000.00312.x 11012885

[27] Dmitriev I, Krasnykh V, Miller CR, Wang M, Kashentseva E, Mikheeva G, et al An adenovirus vector with genetically modified fibers demonstrates expanded tropism via utilization of a coxsackievirus and adenovirus receptor-independent cell entry mechanism. J Virol. 1998;72(12):9706–9713. 10.1128/JVI.72.12.9706-9713.1998 9811704 PMC110480

[28] Giannandrea M, Parks W. Diverse functions of matrix metalloproteinases during fibrosis. Dis Model Mech. 2014;7(2):193–203. 10.1242/dmm.012062 24713275 PMC3917240

[29] Bai W, Wang S, An S, Guo M, Gong G, Liu W, et al Combination therapy of chitosan, gynostemma, and motherwort alleviates the progression of experimental rat chronic renal failure by inhibiting STAT1 activation Oncotarget. 2018;9(21):15498–15511. 10.18632/oncotarget.24125 29643988 PMC5884643

[30] Garcia-Bañuelos J, Siller-Lopez F, Miranda A, Aguilar LK, Aguilar-Cordova E, Armendariz-Borunda J. Cirrhotic rat livers with extensive fibrosis can be safely transduced with clinical-grade adenoviral vectors. Evidence of cirrhosis reversion. Gene Ther. 2002;9(2):127–34. 10.1038/sj.gt.3301647 11857071

[31] Moullier P, Friedlander G, Calise D, Ronco P, Perricaudet M, Ferry N. Adenoviral-mediated gene transfer to renal tubular cells in vivo. Kidney Int. 1994;45(4):1220–1225. 10.1038/ki.1994.162 8007594

[32] Zhu G, Nicolson AG, Cowley BD, Rosen S, Sukhatme VP. In vivo adenovirus-mediated gene transfer into normal and cystic rat kidneys. Gene Ther. 1996;3(4):298–304. 8732161

[33] Thadhani R, Pascual M, Bonventre JV. Acute renal failure. N Engl J Med. 1996;334(22):1448–1460. 10.1056/NEJM199605303342207 8618585

[34] Fujigaki Y. Different modes of renal proximal tubule regeneration in health and disease. World J Nephrol. 2012;1(4):92–99. 10.5527/wjn.v1.i4.92 24175246 PMC3782202

[35] Hynes RO. The extracellular matrix: not just pretty fibrils. Science. 2009;326(5957):1216–1219. 10.1126/science.1176009 19965464 PMC3536535

[36] Ferreras M, Felbor U, Lenhard T, Olsen BR, Delaisse J. Generation and degradation of human endostatin proteins by various proteinases. FEBS Lett. 2000;486(3):247–251. 10.1016/s0014-5793(00)02249-3 11119712

[37] Dong Z, Kumar R, Yang X, Fidler IJ. Macrophage-derived metalloelastase is responsible for the generation of angiostatin in Lewis lung carcinoma. Cell. 1997;88(6):801–810. 10.1016/s0092-8674(00)81926-1 9118223

[38] Ikeda Y, Sun Z, Ru X, Vandenberghe LH, Humphreys BD. Efficient gene transfer to kidney mesenchymal cells using a synthetic adeno-associated viral vector. J Am Soc Nephrol. 2018;29(9):2287–2297. 10.1681/ASN.2018040426 29976586 PMC6115653

