# Supplementary figures and images for: Fibrosis regression is induced by AdhMMP8 in a murine model of chronic kidney injury
Source: PLoS One. 2020 Dec 4;15(12):e0243307. doi: 10.1371/journal.pone.0243307 (PMC7717566; doi:10.1371/journal.pone.0243307)

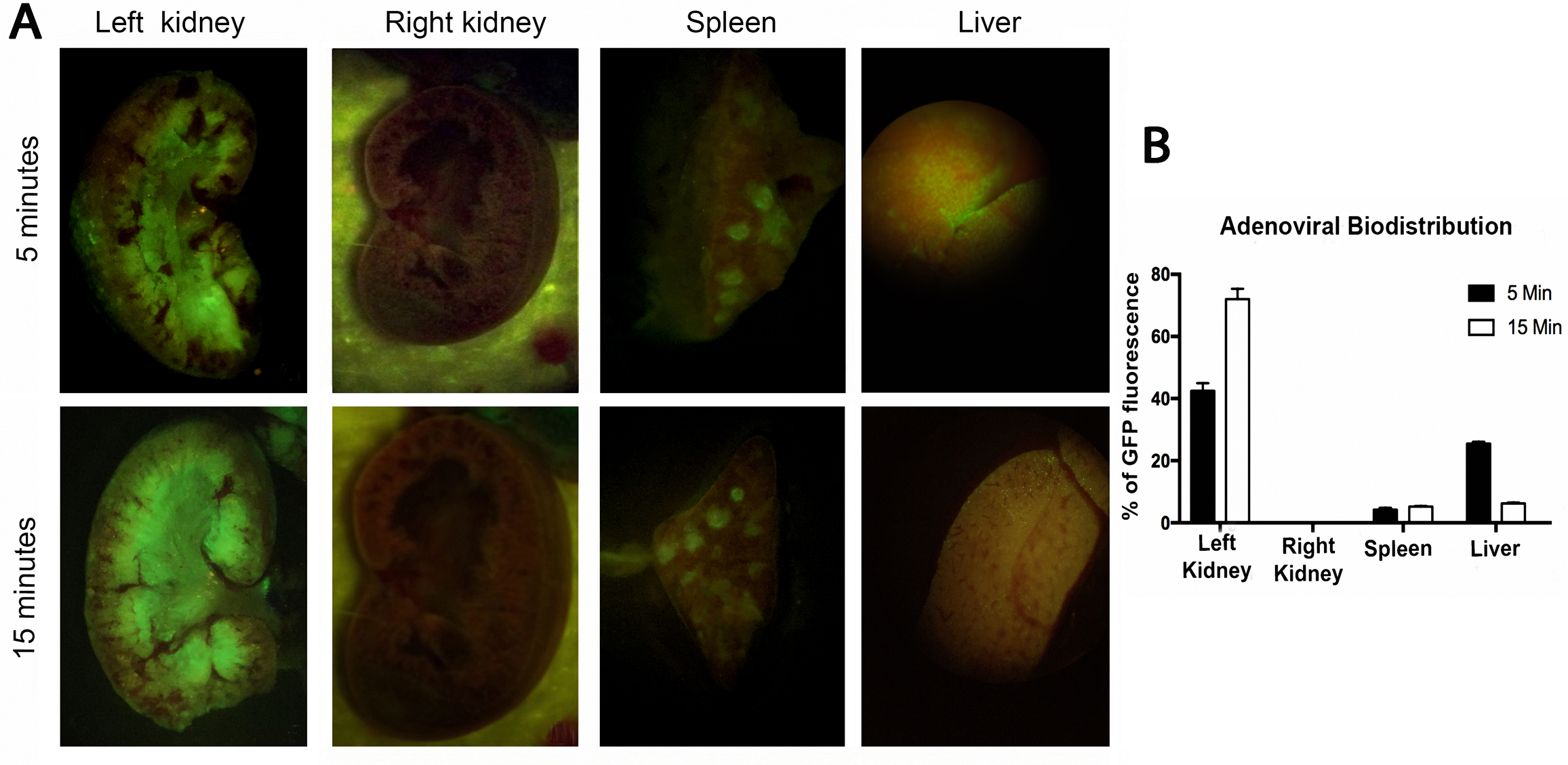

Supplement: S1 Fig — A) Photographs of the reporter gene AdGFP expression in the left kidney, right kidney, spleen and liver after 5 and 15 minutes of administration via retrograde renal vein injections. B) Graph shows percentage of GFP expression according to image analysis mice tissues at 5 and 15 minutes after AdGFP administration. (TIF) [file pone.0243307.s001.tif]

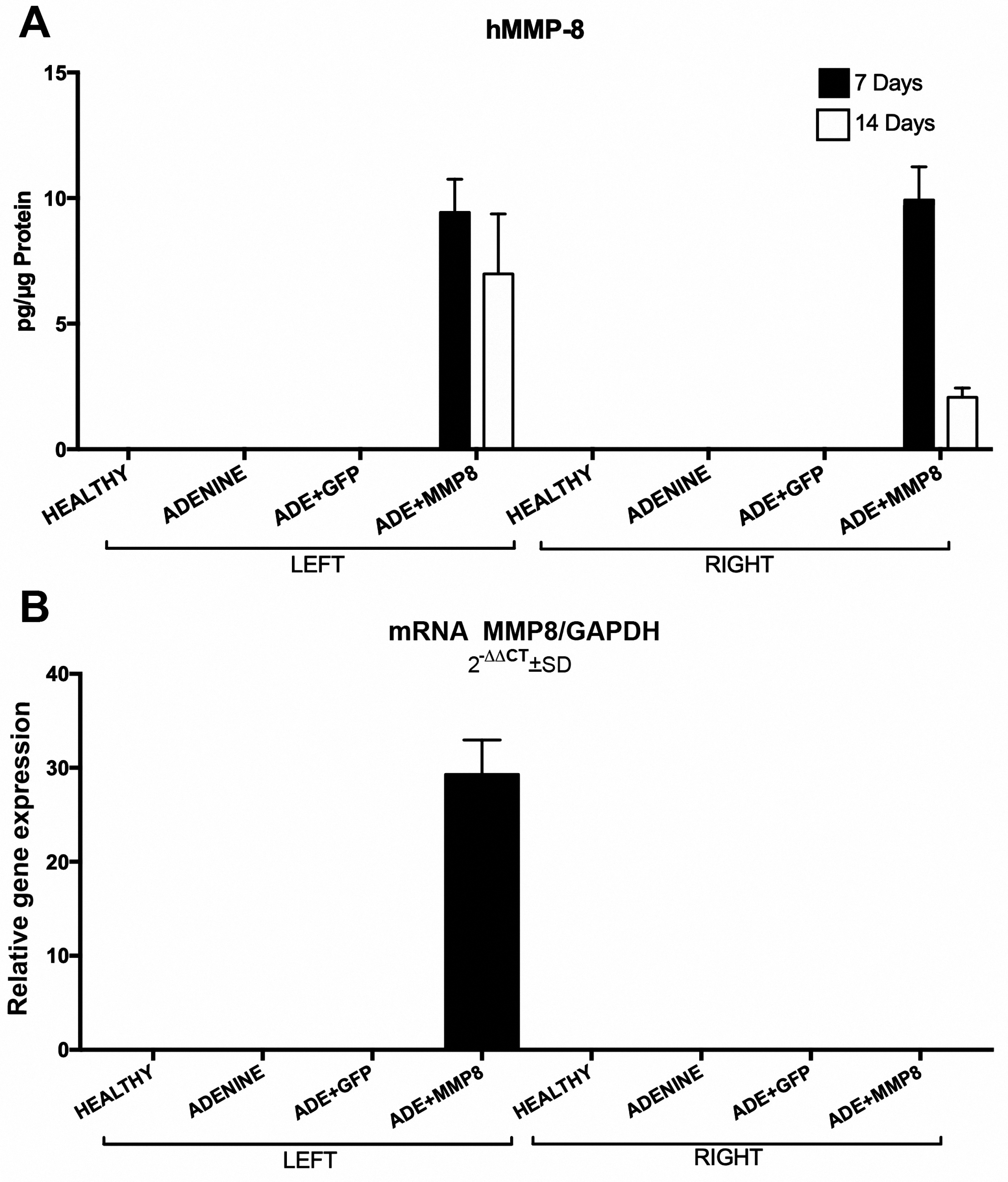

Supplement: S2 Fig — A) Total hMMP8 protein quantification is presented separately in left and right kidney homogenates at day 7 and 14 after adenoviral administration. B) mRNA detection of transduced human MMP8 cDNA in mouse renal samples clearly showed that it is only present in left kidney tissue. (TIF) [file pone.0243307.s002.tif]

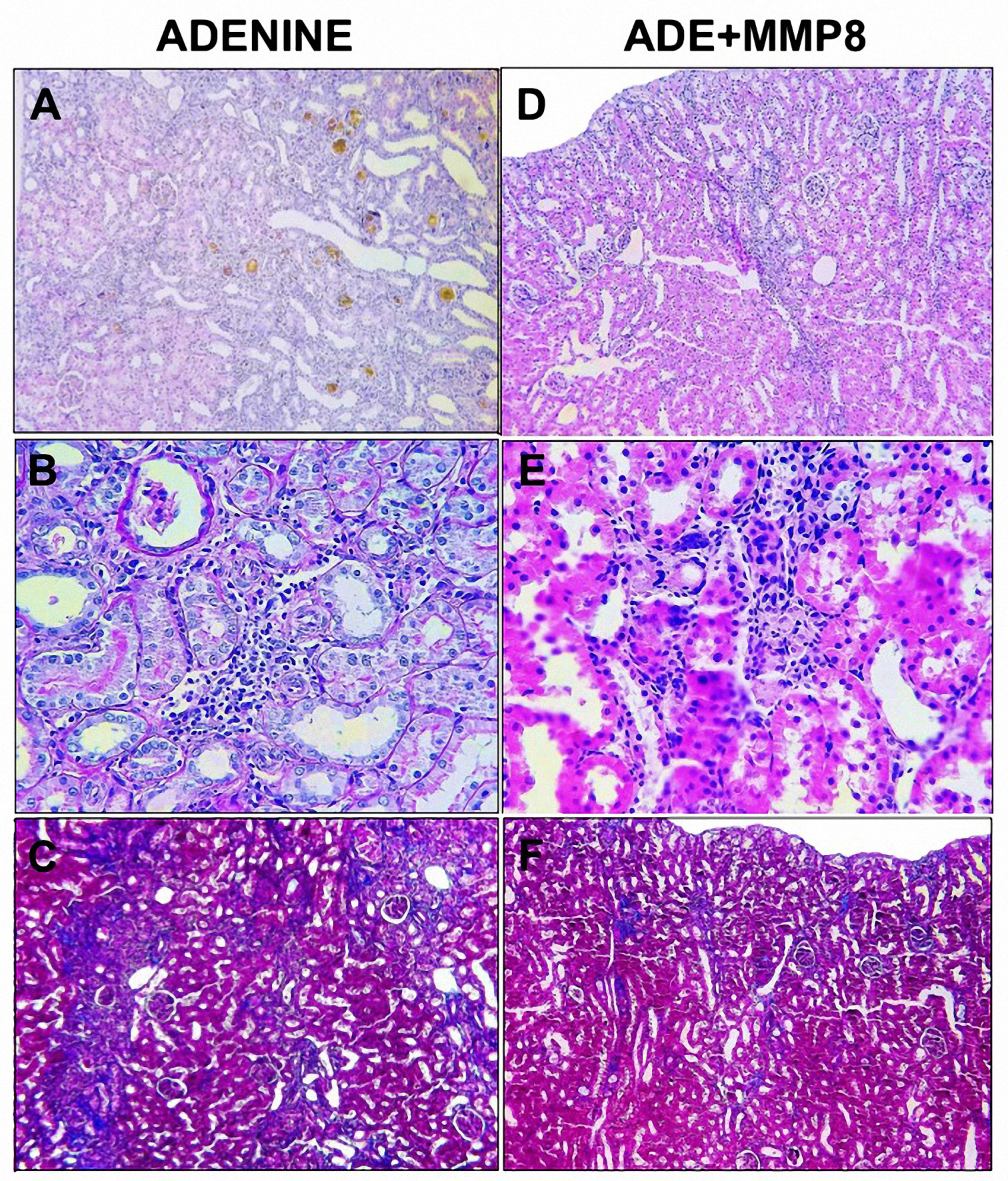

Supplement: S3 Fig — A) Panoramic view of the renal cortex. Adenine crystal deposits in the tubules can be observed and, as response to chronic damaged, tubules dilatation is noticed (HE staining, 10X). B) Tubules with frayed cytoplasm. According to the Banff classification, the focal tubulitis was T2, tissue showed 35% of tubular atrophy and 32.5% of the tissue presented mononuclear inflammatory cells foci (HE staining, 40X). C) Masson staining showed approximately 35% of interstitial fibrosis (10X). D) Panoramic view of the renal cortex; more preserved tissue histology can be observed. The grade of glomerulitis was G1 (HE staining, 10X). E) Renal tubules presented dilatation and frayed cytoplasm. According to Banff classification, focal tubulitis was T1. Tissue showed 15% of tubular atrophy (grade 1: ≤25%) (HE staining, 40X). F) Masson staining showed some areas of interstitial fibrosis. Thus, in the 12.5% of the renal cortex, the interstice space was enlarged in consequence (10X). (TIF) [file pone.0243307.s003.tif]
