# Supplementary material for: Fibrosis regression is induced by AdhMMP8 in a murine model of chronic kidney injury
Source: PLoS One. 2020 Dec 4;15(12):e0243307. doi: 10.1371/journal.pone.0243307 (PMC7717566; doi:10.1371/journal.pone.0243307)
Supplement: S1 Table — (DOCX) [file pone.0243307.s004.docx]

**S1 Table. BANFF CLASSIFICATION**

|  | **Histological Indicators of Renal Damage**  **Rigth kidney** | | |
| --- | --- | --- | --- |
| **Group** | **Interstitial Fibrosis** | **Tubular Atrophy** | **Interstitial Inflammation** |
| **ADENINE** | **2.0** (30.0%±11.54) | **2.0** (35.0%±5.77) | **2.0** (32.5%±2.89) |
| **ADE+AdhMMP8** | **1.0**(12.5%±1.44)* | **1.0**(15.0%±2.89)* | **1.0**(22.5%±1.44)* |

(*p<0.05) As compared to adenine group of rats.
